# Anatomy and Histology of Sensorimotor Connections Between the Facial and Trigeminal Nerve in the Buccinator Muscle

**DOI:** 10.1002/hed.27990

**Published:** 2024-11-07

**Authors:** Ahneesh J. Mohanty, Joep A. F. van Rooij, Renée M. L. Miseré, Arno Lataster, Shai M. Rozen, René R. W. J. van der Hulst, Stefania M. H. Tuinder

**Affiliations:** ^1^ Department of Plastic and Reconstructive Surgery University of Texas Southwestern Medical Center Dallas Texas USA; ^2^ Department of Plastic, Reconstructive, and Hand Surgery Maastricht University Medical Center Maastricht Netherlands; ^3^ Department of Anatomy and Embryology, Faculty of Health, Medicine and Life Sciences Maastricht University Maastricht Netherlands

**Keywords:** buccinator muscle, facial nerve, histology, interconnections, trigeminal nerve

## Abstract

**Background:**

Despite indications of a close interaction between the trigeminal (CN V) and facial nerve (CN VII) within the buccinator muscle, a combination of anatomical dissection and histological analysis has not been reported.

**Methods:**

Five formalin‐fixed and fresh‐frozen hemifaces were dissected to reveal the buccal fat pad, the buccinator muscle, and anastomotic connections between CN V and CN VII within it. Samples were taken for histological processing and immunostaining.

**Results:**

Branches of CN V and CN VII formed pronounced sensorimotor anastomotic connections in and surrounding the buccinator muscle. These findings were histologically evident with close intramuscular coupling of sensory and motor fibers. There was an evident but gradual shift from motor to sensory fibers in the interconnections when analyzing them from the side of CN V toward the side of CN VII and vice versa.

**Conclusions:**

These results further elucidate connections between CN V and CN VII and their possible role in proprioception of the facial muscles.

## 1 Introduction

The trigeminal nerve (CN V) is the largest of the cranial nerves and provides both sensory as well as motor function. Its three branches, the ophthalmic (CN V_1_), maxillary (CN V_2_), and mandibular (CN V_3_) nerves innervate the facial skin for different forms of sensation and innervate the muscles of mastication for processing of food [1]. The facial nerve (CN VII) also contains a mix of motor and sensory fibers. It innervates the facial muscles and the stapedius muscle, provides sensation to the external auditory meatus, the tympanic membrane and the pinna of the ear, provides function of taste to the anterior two‐thirds of the tongue, and parasympathetic fibers supply the lacrimal gland and glands in the oral cavity [2]. Despite their differences in function, there is a relationship between the two nerves in some way, as anastomoses between CN V and CN VII have abundantly been found all around the face in previous anatomical studies [3].

With CN VII innervating the facial muscles and CN V innervating the muscles of mastication, the buccinator is unique in that it is the only facial muscle involved in mastication, while being innervated by CN VII [4, 5]. The buccinator, derived from the second pharyngeal arch, forms the lateral wall of the oral cavity with horizontal fibers arising from the pterygomandibular raphe and interdigitating anteriorly with fibers of the orbicularis oris at the modiolus [6]. Its primary role is to compress the oral mucosa against the teeth in order to keep food between the tooth surfaces, as well as assisting with sucking, blowing, and speech [5]. Electromyographic studies have shown modulation of masticatory activity of the buccinator muscle, in which the muscle relaxes and contracts in response to consistency and quantity of food [7, 8]. These findings suggest a close interplay of CN V sensory and CN VII motor feedback which orchestrate the function of this muscle [9].

The buccinator muscle directly underlies a plexiform‐like network of neural anastomoses between CN V and CN VII located within the buccal fat pad [10]. These interconnections between the two nerves are present at more locations in the face, such as the temporal, ocular, and oral region [11, 12, 13, 14, 15, 16]. In their anatomical and histological study, Hwang et al. showed that merging branches of CN V and CN VII share the same epineurium, further suggesting a connection between the two [11]. However, a shared epineurium does not mean a shared perineurium and it does not prove that they truly interconnect, but merely that they follow a similar path [12, 13]. Evidence of an interplay between the nerves is mounting. In spite of this, a combination of anatomical dissection, immunostaining, and histological analysis of anastomoses has not previously been done.

Accordingly, we performed this exploratory study to evaluate the anatomical and histological characteristics of the buccinator muscle in order to better understand the possible interactions between CN V and CN VII.

## 2 Materials and Methods

### 2.1 Dissection of Anatomic Specimens

Anatomic specimens for this study were obtained from the Department of Anatomy and Embryology, Faculty of Health, Medicine and Life Sciences, Maastricht University, Maastricht, the Netherlands. A handwritten and signed codicil expressing consent for donation was obtained for each specimen. Institutional review board approval was not required.

Three formalin‐fixed hemifaces from two female anatomic specimens (75 and 99 years) and two fresh‐frozen hemifaces from one female anatomic specimen (95 years) were dissected to evaluate the anatomic course of CN V and CN VII. None of the specimens had a history of facial surgery. The fresh‐frozen specimens were stored at −30°C and thawed at room temperature prior to the first dissection. All specimens were preserved in cold formalin (4°C) between the sessions. The dissections were performed under loupe magnification.

**FIGURE 1:**
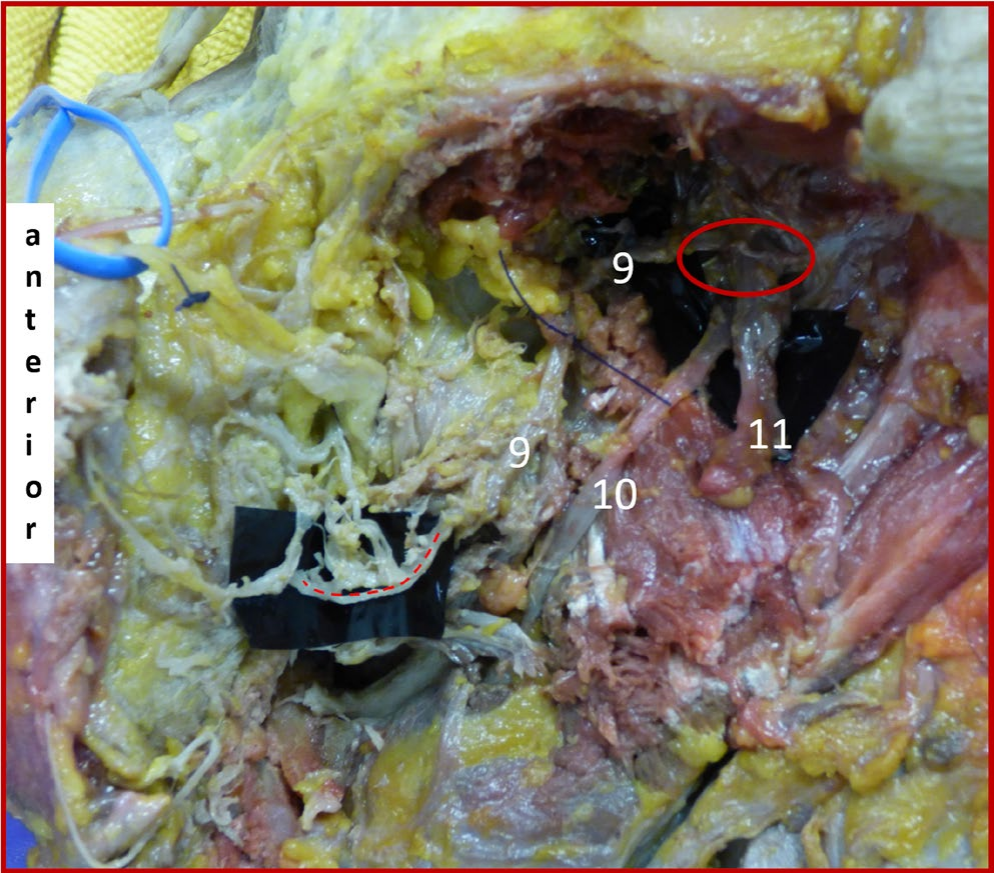
Formalin‐fixed left hemiface. Dissection of trigeminal (CN V) and facial nerve (CN VII) anastomoses within the buccal fat pad after mandibular resection and anterior reflection of parotid gland following masseter dissection and cutting through the main facial nerve trunk. 9 = buccal nerve originating from mandibular branch of trigeminal nerve (CN V_3_); 10 = lingual nerve; 11 = inferior alveolar nerve (cut); red circle = exit of mandibular branch of the trigeminal nerve at the foramen ovale; red dashed line = anastomosis between buccal nerve (CN V) and CN VII branches. [Color figure can be viewed at wileyonlinelibrary.com]

A preauricular skin incision was made and a composite skin‐SMAS (superficial musculoaponeurotic system) flap was dissected off the parotid fascia. At the anterior edge of the parotid gland, CN VII branches were identified, tagged with vessel loops, and dissected distally into their point of entry into the mimetic muscles. The parotid duct, facial artery, and facial vein were identified and tagged with vessel loops. Proximal dissection reached the stylomastoid foramen. The main facial nerve trunk was cut there and reflected anteriorly together with the parotid gland, remaining in a plane superficial to the masseter muscle. After the mandibular nerve was identified at the mandibular notch, the masseteric muscle was detached from the mandible and lifted cranially toward the zygomatic arch, leaving a vertical strip of muscle to prevent damage to the connections between the buccal nerve (CN V) and branches of CN VII. A vertical osteotomy through the mandibular body was performed midway between the mental foramen and the mandibular angle. The posterior mandibular body and ramus were removed after detaching the temporalis muscle from the coronoid process, disinserting the lateral pterygoid muscles from the condylar neck and temporomandibular joint and the medial pterygoid muscle from the medial mandibular surface, and finally disarticulating the condyle from the mandibular fossa. This required cutting the inferior alveolar nerve as well as the distal branches of the mandibular nerve to the temporalis and pterygoid muscles. The lingual nerve and the buccal nerve (CN V) were identified and dissected proximally toward their exit from the foramen ovale. Branches of CN VII and the buccal nerve (CN V) were then followed distally to the buccal fat pad (Figure 1). Within this space, anastomoses between the buccal nerve (CN V) and CN VII were identified, meticulously dissected, and resected en bloc in order to preserve proper orientation for further histological analysis. Additionally, random biopsies of the buccinator muscle were taken during the first dissection and prepared for comparative histological analysis. A step‐wise description of the dissection, including photographs, can be found in the Supporting Information S1.

### 2.2 Histological Analysis

All histological analyses were performed using hematoxylin and eosin (H&E) and immunostaining. Excised specimens were fixed with cold 10% phosphate buffered formalin. Subsequently, specimens were paraffin embedded and then manually sectioned with a microtome (Leica RM2245, Leica Biosystems Nussloch Germany) to obtain 5 μm‐thick paraffin sections. H&E staining was performed to provide a general overview of the tissue morphology. Tyrosine hydroxylase (TH) and vesicular acetylcholine transporter (VAChT) labeling were used for immunohistochemical identification of nerve fiber types. Histological images were obtained with an Olympus BX61 versus scanning microscope and staining intensity was quantified using ImageJ software [17]. All histological images were converted to grayscale for standardization. ImageJ quantifies the greyscale intensity of each pixel within a given region of interest. Regions of interest in the histological images were defined as an individual nerve fascicle for each fascicle present. The median gray scale values of each fascicle were taken and plotted on a graph. Statistical analysis was performed by a linear regression model in GraphPad Prism for Windows (GraphPad Software, Boston, Massachusetts USA, www.graphpad.com), analyzing the signal intensity in relation to the length along the CN V to CN VII interconnections.

## 3 Results

### 3.1 Macroscopic Anatomy

In the five hemifaces, the buccinator muscle was found to be reliably innervated through a plexiform‐like network of anastomoses between the buccal nerve (CN V), coming from the mandibular nerve (CN V_3_), and the buccal and zygomatic branches of CN VII found within the buccal fat pad. On average, two CN VII branches were found to contribute to this plexus, which then further arborized into anastomotic connections with the buccal nerve (CN V). These connections were then found to penetrate into the buccinator muscle.

### 3.2 Microscopic Anatomy

**FIGURE 2:**
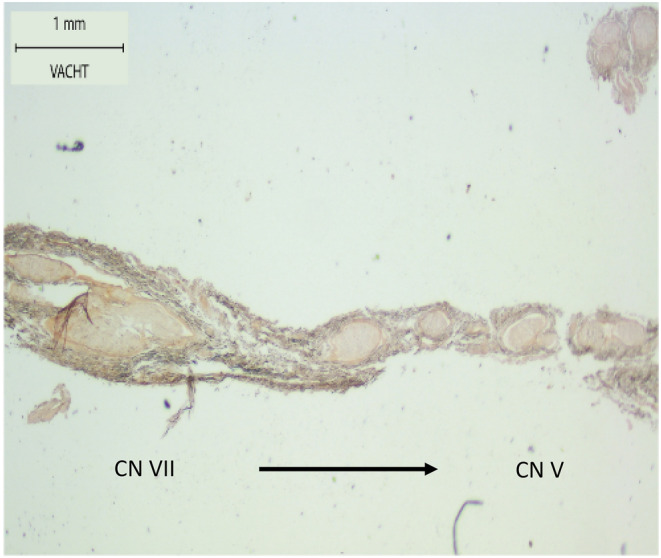
Vesicular acetylcholine transporter (VAChT; brown color stain) immunostaining of a CN VII—CN V anastomosis in sagittal view as it arises from CN VII (left side of figure) and connects to the mandibular nerve (CN V_3_) (right side of figure). The nerves can be seen as large circles in the tissue, surrounded by epineurium. The patched nature of the nerves in this sample is caused by sectioning in the sagittal plane. A slight color gradient can be seen jumping from the left to the right nerve sections, indicating a decrease in VAChT staining (= motor fibers) when moving more toward CN V (black arrow). [Color figure can be viewed at wileyonlinelibrary.com]

**FIGURE 3:**
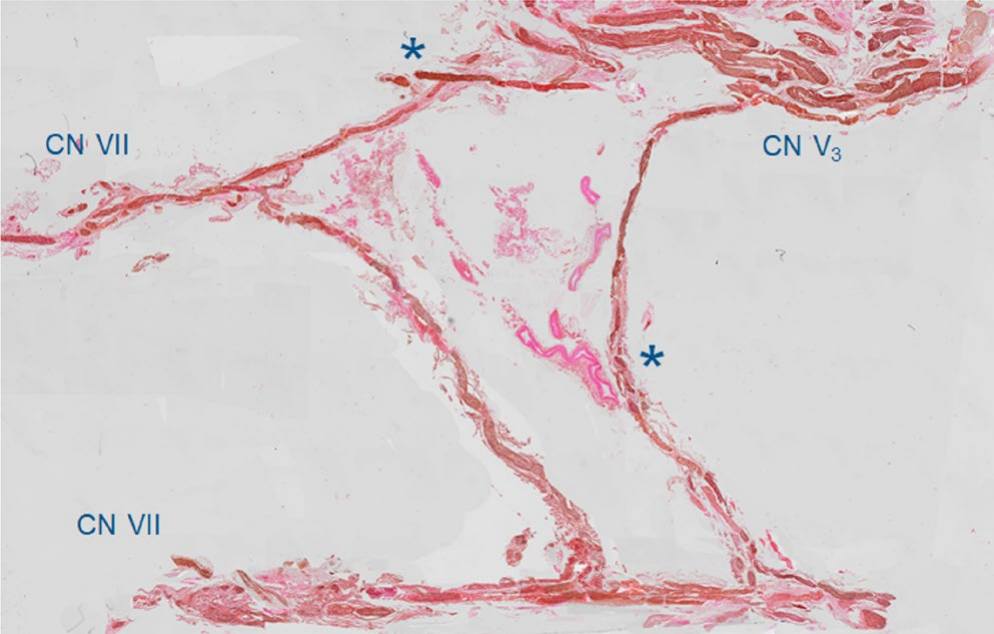
Sagittal histological section of the CN V—CN VII plexus after hematoxylin and eosin (H&E) staining. Asterisks denote anastomoses between CN V and CN VII. [Color figure can be viewed at wileyonlinelibrary.com]

Each hemiface contributed three anastomotic connections between CN V and CN VII, resulting in a total of 15 anastomoses for histology. Histological analysis and immunostaining of these anastomotic connections was performed. The nature of the nerve fibers was characterized in three ways based on the immunostaining. First, it was characterized through the absence of TH immunostaining, precluding autonomic contributions. Second, through the presence or absence of VAChT immunostaining, with positivity suggesting the presence of motor fibers. And third, through dual absence of TH and VAChT staining, suggesting the presence of sensory fibers. Negativity of immunostaining causes pale tissue on histological images. Positivity of TH is shown by brown staining of tissue. Positivity of VAChT is also shown by brown staining of tissue. These investigations revealed sensory fibers arising from the trigeminal contribution and motor fibers arising from the facial contribution, resulting in a coalescing of sensory and motor fibers within these anastomotic connections. This can be seen on the color gradient in Figure 2, where staining of the nerve fibers with VAChT gradually decreases when moving more toward CN V and vice versa. This denotes a gradual decrease in motor fibers moving from the side of CN VII to the side of CN V and vice versa. Subsequently, these connections gave rise to perforating branches into the muscle which were visible on histological analysis (Figure 3).

**FIGURE 4:**
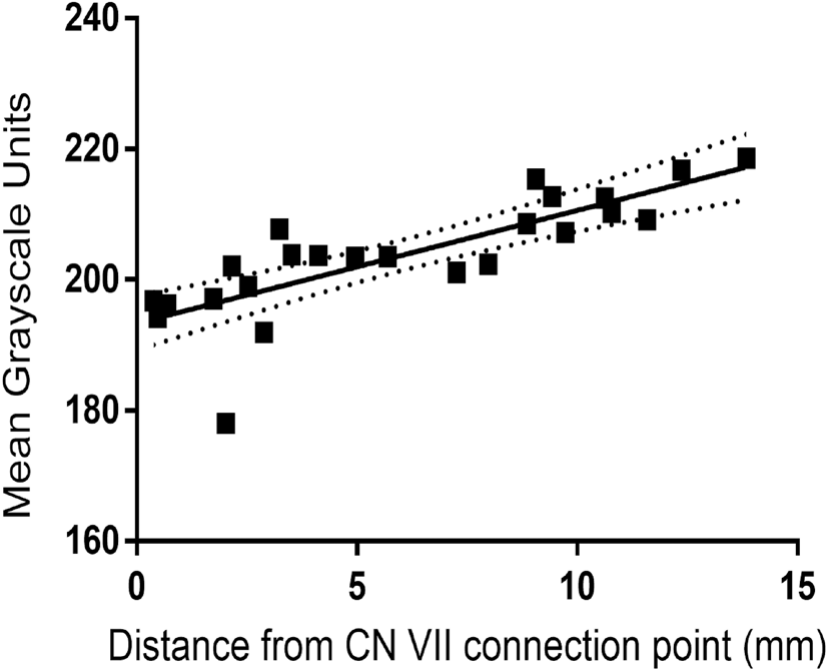
Quantification of mean vesicular acetylcholine transporter (VAChT) staining intensity along the CN V—CN VII anastomosis as distance increases from the CN VII connection point.

The observation of a close relationship between the sensory and motor fibers was further supported by quantification of staining intensity in the anastomotic connections (*n* = 15; 5 hemifaces each contributing 3 anastomotic connections) using ImageJ software (Figure 4) [17]. This indicates an admixture of sensory and motor fibers occurring throughout the connection, with greater proportions of sensory or motor fibers occurring closer to their respective origins.

**FIGURE 5:**
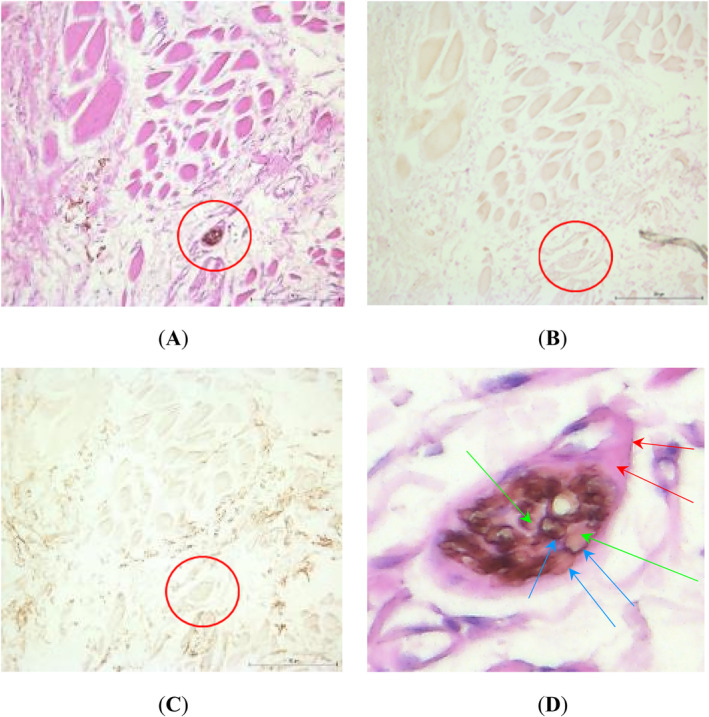
A–D Staining of buccinator muscle histological sections using (A) hematoxylin and eosin (H&E) staining; (B) tyrosine hydroxylase (TH) immunostaining, showing no staining of the tissues in or surrounding the muscle fibers; (C) vesicular acetylcholine transporter (VAChT, brown) immunostaining, showing diffuse positive staining for motor fibers in the buccinator muscle; (D) image enlargement of H&E stained nerve from (A), scale 1.0 cm: 35 μm. Imaged at 10× magnification. Red circle = section of a larger nerve in the buccinator muscle; red arrows = epineurium; blue arrows = perineurium; green arrows = inside perineurium, where individual axons surrounded by endoneurium lie (individual axons are not visible due to restrictions in tissue quality). Based on the absence of VAChT and TH staining, the circled nerve is most likely a sensory nerve coming from the buccal nerve (CN V). [Color figure can be viewed at wileyonlinelibrary.com]

The same immunostaining algorithm to characterize nerve fibers was employed for microanatomical analysis of buccinator muscle specimens. Within the buccinator muscle, motor nerve fibers were found to be closely juxtaposed to sensory fibers (Figure 5A–D). In the buccinator muscle samples, a mixture of sensory and motor fibers can be seen as the larger nerve is negative for TH and VAChT, indicating a sensory origin. Also, smaller surrounding fibers are positive for VAChT, indicating a motor origin.

## 4 Discussion

The presence of plexiform connections between terminal rami of the CN V and CN VII is well known and has been described in anatomical textbooks for decades. However, increasing knowledge is gathered on the microscopic anatomy and histology of these interconnections. This study aimed to provide further insight into the complicated sensorimotor interaction between the two nerves. Our findings suggest that the buccinator muscle receives rich sensorimotor innervation through plexus‐like connections between branches of the buccal nerve (CN V) and buccal branches of CN VII, resulting in close sensory and motor coupling. Apart from the connections visible during dissection, immunostaining has shown that the amount of motor fibers in the anastomoses gradually increases when moving from CN V toward CN VII, while dual negative immunostaining suggested the presence of sensory fibers and vice versa. The close relationship between CN V and CN VII is further elucidated by the mixture of sensory and motor nerve fibers found in biopsies of the buccinator muscle.

### 4.1 Macroscopic Connections

Trigeminofacial anastomoses are found throughout the face: at the frontal and temporal regions, in the midface, and also in the lower third of the face [6, 18]. At the frontal and temporal regions, anastomoses can be found between the zygomaticotemporal or supraorbital nerve (CN V) and temporal branches of CN VII [6, 14, 15, 19, 20, 21].

In the midface, branches of the infraorbital nerve (CN V) form direct connections with CN VII running toward the facial muscles as both nerves run into the infraorbital plexus [6, 22, 23, 24]. A critical zone of the infraorbital plexus lies in a 36‐mm diameter circle, with its center 22 mm inferior from the infraorbital foramen. In this circle, approximately 96% of all merging points between the superior labial branch and CN VII are located, which clinicians should keep in mind during procedures in the surrounding anatomical areas [11].

In the lower face, it is often the buccal nerve (CN V) or the mental nerve (CN V) that is involved in anastomoses with the branches of CN VII, such as the marginal mandibular and cervical branch, but the buccal and mental nerve also communicate with each other [6, 25]. Anastomoses with the mental nerve often happen in close proximity of the mental foramen [12, 13, 25].

Overall, the presence of communications throughout the face varies, the location varies, and also the amount of contact they have, ranging from only touching of the CN V and CN VII branches to truly connecting [24, 26]. There seems to be a vast anatomical network in the face of communications between the sensory CN V and the motor CN VII of both large and small nerve fibers, with the maxillary nerve (CN V_2_) having the highest frequency of communication with CN VII [3]. Knowledge of the macroscopic anatomy and location of these anastomoses can be of importance to surgeons to prevent injury during surgery and to keep the connections intact, because they might have functional importance, on which we will elaborate further below.

Evaluation of CN V and CN VII anastomoses specifically in the buccinator muscle itself is rather unique. Tohma et al. found anastomoses surrounding the buccinator muscle. They found a consistent branch of the buccal nerve (CN V), the so‐called “communicating buccal nerve (CBN),” to emerge at the outer fascia of the anterior portion of the buccinator muscle, proceeding and intermingling across the anterior surface of the muscle [10]. Here, it tightly surrounds and follows the course of the facial vein. Anterior twigs of the CBN supplied the muscles at the area lateral to the mouth, which was also found by others [27]. The CBN originates from the buccal nerve (CN V) and joins CN VII. Shimada et al. is the only study who investigated the connections inside the buccinator itself and found branches of the buccal nerve (CN V) and the buccal branches of CN VII to connect [18]. Our study had similar findings, but added microscopic and histological evaluation.

### 4.2 Microscopic Connections

Apart from macroscopic investigation of connections, microscopic and histological analysis can provide a more comprehensive view of the trigeminofacial relationship. In humans, Hwang et al. have performed several anatomical and histological studies on the communications between the supraorbital nerve, mental nerve, and infraorbital nerve (CN V), and branches of CN VII [11, 12, 14]. After H&E staining of the horizontal branch of the supraorbital nerve (CN V) and the temporal branch (CN VII), they found the epineurium of CN V and CN VII to fuse in the anastomoses, while keeping a separate perineurium [14]. This suggests that the nerves follow the same path, but do not truly fuse. However, they have only analyzed one sample of a neural cross‐section in the frontal plane and used only H&E staining, therefore not being able to show the nature of the nerve fibers. In our study, we have analyzed sagittal/longitudinal sections and have used immunostaining with VAChT and TH to differentiate between motor and sensory fibers. Accordingly, we have been able to show an increase in the number of motor fibers and a decrease in the number of sensory fibers when analyzing staining intensity of VAChT in the anastomoses from the side of CN V toward the side of CN VII (and vice versa). This suggests intercommunications between the two at microscopic level as well.

Comparable results to the earlier mentioned study by Hwang et al. were found by Iwanaga et al. when microscopically investigating connections between the mental nerve (CN V) and the marginal mandibular branch (CN VII) [13]. Masson‐trichrome staining was used and two or more separate perineuria were found within one epineurium. Hwang et al., however, found one epineurium and poor perineurium after fusion of the infraorbital nerve with branches of CN VII [11].

### 4.3 Function of the Trigeminofacial Anastomoses

Still, the question remains as to the function of the trigeminofacial anastomoses [28]. Interaction between CN V and CN VII has been suggested for a long time. According to Baumel, the trigeminofacial connections may occur at three levels: centrally within the brain stem, as deep facial communications, or as superficial facial communications [29]. Evidence for the role of CN V in facial proprioception is growing [29, 30, 31]. The CN VII innervated facial muscles are unique, because they do not possess the conventional mechanisms of proprioception like the skeletal muscles in the rest of the body do [32, 33]. Hence, cutaneous stretching and therefore connections between CN V and CN VII are thought to play an important role in the proprioceptive regulation of the face, just as cutaneous afferents are essential to proprioception of the fingers [34, 35, 36, 37]. In that case, the fibers of CN V may operate for afferent proprioceptive signaling, while the CN VII fibers return motor signaling after processing of information [20]. Also, a recent experimental study in rats has shown altered movement of the whiskers after complete unilateral transection of all CN V branches, while leaving CN VII intact [38]. Transcranial magnetic stimulation (TMS) studies have shown inhibition of facial motor function after stimulation of trigeminal sensory afferents and findings suggest there are different roles for cutaneous and muscular afferents in regulating facial muscle activity, because they distinctly influence excitability of the facial motor cortex [39, 40, 41]. These findings further substantiate a possible functional connectivity between the two nerves.

Furthermore, earlier studies have shown that patients may regain motor function spontaneously after transection of their CN VII [18, 42, 43, 44]. Interestingly, during reoperation of four of their subjects, Conley et al. found that regeneration of the CN VII ends did not happen [45]. This indicates that the motor impulses may have followed a different path than usual, and accordingly Martin and Helsper reported that voluntary motor impulses may find their way via the brain through the trigeminofacial connections to activate the facial muscles. They also hypothesized that the connections must be there for some particular functional reason [44]. In some cases, transsagittal reinnervation by fibers from the opposite CN VII has been described, especially surrounding the mouth [29]. However, despite multiple hypotheses, the true mechanism behind the spontaneous reinnervation remains largely unknown. Recently, Terehsenko et al. have shown that denervation of rodent facial muscles was followed by a spontaneous reinnervation by autonomic nerve fibers via the infraorbital nerve (CN V) [46]. A finding which might elucidate a comparable mechanism in the spontaneous recovery of motor function in humans. However, description of spontaneous recovery remain scarce and the role of the trigeminal nerve in recovery after facial palsy through spontaneous reinnervation seems to be the exception rather than the rule.

The exact clinical implications of damaging the connections remains however uncertain, but as disruption could alter its integrity and functioning, the head and neck surgeon may beware of and preserve the anastomoses [47].

### 4.4 Limitations

Our study has some limitations. First, the golden standard for retrieving tissue for histology is to take samples as fresh as possible. In some anatomic specimens, the tissues were not fresh‐frozen, but formalin‐fixed. This could possibly influence the tissue quality. Due to this, we were not able to visualize the individual axons with their endoneurium. The two fresh‐frozen hemifaces were thawed at room temperature prior to the first dissection. This was followed by fixation through immersion in cold formalin, after which the rest of the dissection was performed. Nerve samples were taken during all three dissection sessions. Second, due to the histological section of the samples in the sagittal plane, we were unable to clearly visualize the perineurium which is easily disrupted during sectioning. Future studies should focus on more elaborate immunostaining and higher magnification microscopy to further investigate the microscopic anatomy of the CN V and CN VII bridges.

## 5 Conclusion

Connections between CN V and CN VII are present all around the human face and they have been studied numerous times by a large number of authors. However, there remains much to be unknown about the nature and function of these connections, meaning much needs to be further elucidated. One of these unknowns is the relationship between CN V and CN VII in the innervation of the buccinator muscle. The buccinator muscle is unique in that it receives robust sensory innervation from CN V and motor innervation from CN VII. Our study has shown that there is a gradual transition from sensory to motor nerve fibers moving from CN V to CN VII, suggesting communication and possibly reciprocal influence between the two.

## Author Contributions

**Ahneesh J. Mohanty:** conceptualization, methodology, validation, formal analysis, writing – original draft preparation, writing – review and editing, visualization, approval of manuscript. **Joep A. F. van Rooij:** conceptualization, methodology, validation, writing – original draft preparation, writing – review and editing, visualization, approval of manuscript. **Renée M. L. Miseré:** conceptualization, methodology, validation, writing – original draft preparation, visualization, approval of manuscript. **Arno Lataster:** conceptualization, methodology, validation, resources, writing – review and editing, visualization, supervision, approval of manuscript. **Shai M. Rozen:** conceptualization, methodology, writing – review and editing, supervision, approval of manuscript. **René R. W. J. van der Hulst:** conceptualization, writing – review and editing, supervision, approval of manuscript. **Stefania M. H. Tuinder:** conceptualization, methodology, validation, writing – original draft preparation, writing – review and editing, visualization, supervision, approval of manuscript. All authors have read and agreed to the published version of the manuscript.

## Ethics Statement

The authors have nothing to report.

## Consent

A handwritten and signed codicil expressing consent for donation was obtained for each specimen.

## Conflicts of Interest

The authors declare no conflicts of interest.

## Supporting information


**Supporting Information S1.**: A step‐wise description of the dissection, including photographs, can be found in the Supporting Information.

## Data Availability

The data that support the findings of this study are available from the corresponding author upon reasonable request.
