# Supplementary material for: Anatomy and Histology of Sensorimotor Connections Between the Facial and Trigeminal Nerve in the Buccinator Muscle
Source: Head Neck. 2024 Nov 7;47(3):928–35. doi: 10.1002/hed.27990 (PMC11816563; doi:10.1002/hed.27990)
Supplement: Supplementary file 1 — Supporting Information S1.: A step‐wise description of the dissection, including photographs, can be found in the Supporting Information. [file HED-47-928-s001.docx]

**SUPPLEMENTARY MATERIALS –
STEP-WISE DESCRIPTION OF ANATOMICAL DISSECTION**

Dissections were performed under loupe magnification. Three formalin-fixed and two fresh-frozen hemifaces were dissected. The formalin-fixed hemifaces were formalin-fixed beforehand. The fresh-frozen hemifaces were immersion fixed in the course of the dissection session, as can be seen in between the sessions below.

***First session – fresh-frozen hemiface***

1. Thawed hemiface was put on ice.
2. Dissection of preauricular skin-SMAS flap off parotid fascia (Picture S1).
3. Identification of the branches of the facial nerve at the anterior edge of the parotid gland and tagging of the branches with vessel loops (Picture S1).
4. Dissection of facial nerve branches into their entry point to the mimetic muscles (Picture S2).
5. Identification of the parotid duct and tagging with a vessel loop (Picture S2).
6. Identification of the facial artery and vein and tagging with a vessel loop.
7. Following of the identified facial nerve branch proximally until foramen stylomastoideum with complete identification of the facial nerve trunks (Picture S3).
8. Cutting of the main trunk of the facial nerve (Picture S4).
9. Anterior reflection of the parotid gland and facial nerve, remaining in a plane above the masseter muscle (Picture S3 + S5 + S6).
10. Identification of both branches of the masseteric nerve at mandibular notch, between the coronoid process and the mandibular condyle (Picture S7).
11. Detachment of the masseter muscle from the mandible, leaving a lengthwise strip of muscle (to avoid damage to connections between the buccal nerve and the facial nerve) and lifting it cranially to the zygomatic arch (Picture S7).
12. Storage of specimen in cold formalin (4°C / 39.2°F).

***Second session – partial formalin already***

1. Performing of vertical osteotomy through mandibular body, between mental foramen and mandibular angle and oblique osteotomy at condylar neck (alternative option is direct exarticulation of condylar process)
2. Detachment of temporalis muscle from the coronoid process (Picture S8).
3. Detachment of lateral pterygoid muscle from condylar neck and temporomandibular joint (Picture S8).
4. Detachment of medial pterygoid muscles (lateral and medial head) from medial mandibular surface (Picture S8).
5. Disarticulation of the condyle from temporomandibular joint and remove sawn bone. This requires cutting the inferior alveolar nerve and distal branches of the mandibular nerve to the temporalis muscle and pterygoid muscles (Picture S9).
6. Identification of the lingual nerve and buccal nerve and exploration of both nerves proximally to their exit of the foramen ovale (Picture S9).
7. Further following of the facial nerve branches anteriorly to Bichat’s fat pad (Picture S9).
8. Storage of specimen in cold formalin (4°C / 39.2°F) (Picture S9).

***Third session – formalin***

1. Careful identification within space of Bichat’s fat pad of anastomoses between buccal nerve (from trigeminal nerve) and facial nerve (Picture S10 + S11).
2. Meticulous dissection of anastomoses and en bloc resection in order to preserve proper orientation for further histological analysis (Picture S10 + S11).
3. Mounting of en bloc resection on tempex in the same orientation as in the whole specimen (Picture S12).
4. Floating upside-down fixation of resection in 10% phosphate buffered formalin.


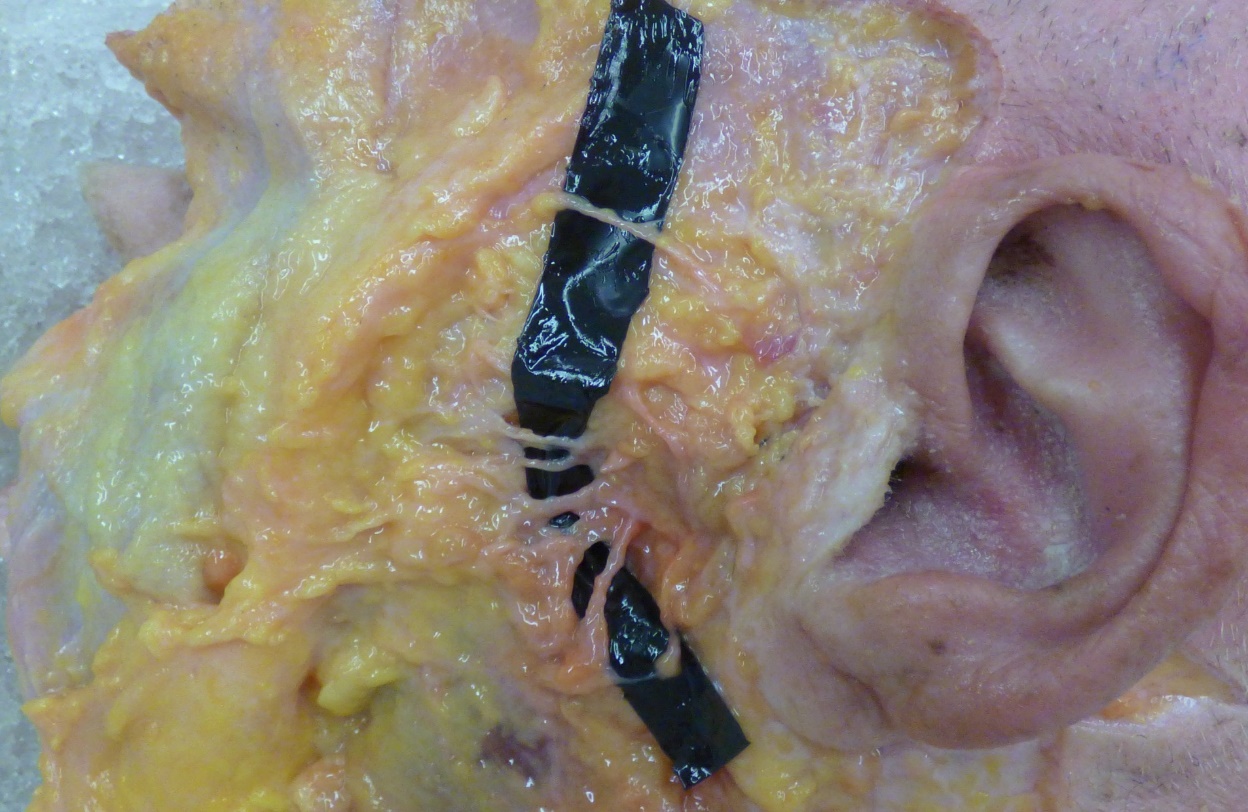


1

2

3

4

**2+3**

**Picture S1** Fresh-frozen left hemiface after steps 2 and 3 of dissection. 1 = zygomatic branch of facial nerve; 2 + 3 = buccal branches of facial nerve; 4 = marginal branch of facial nerve


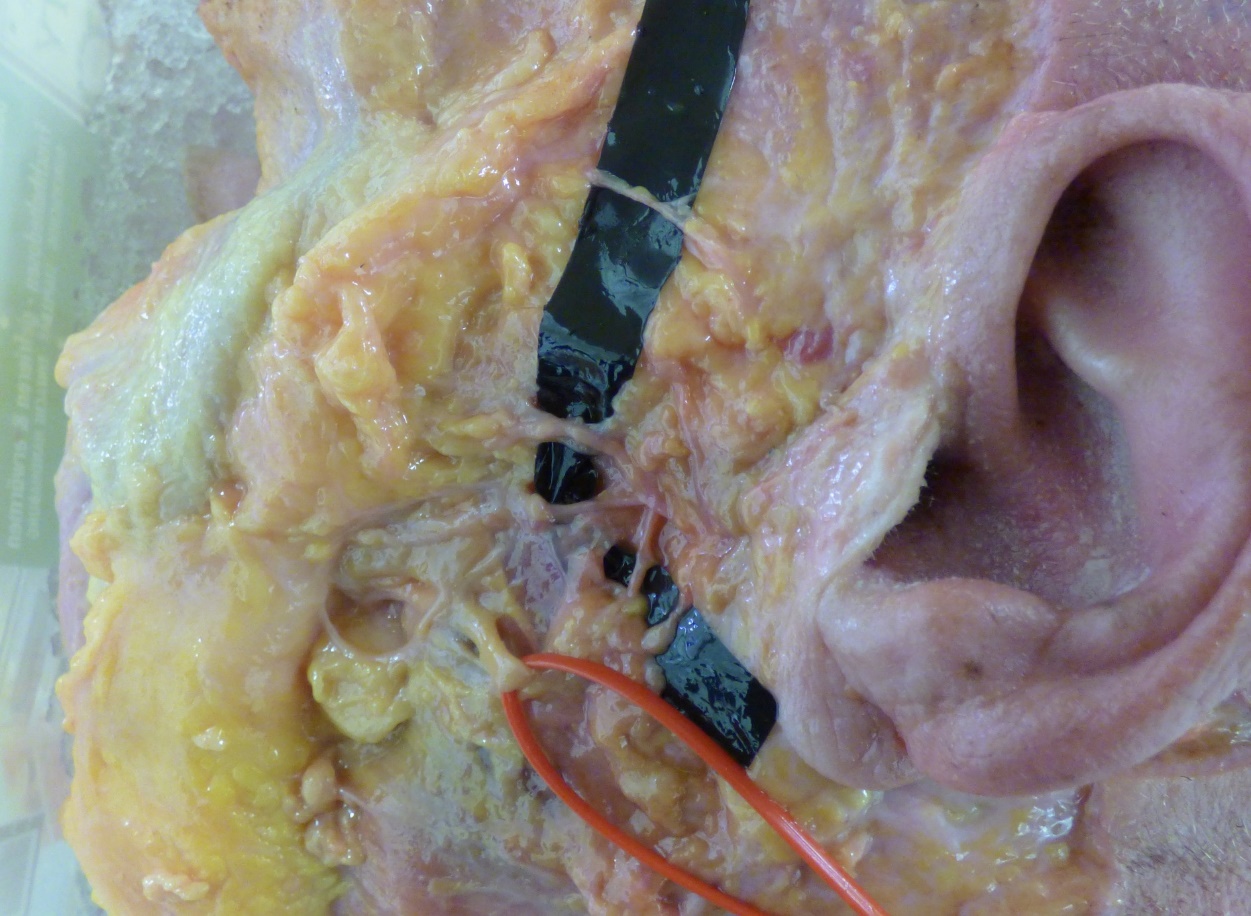


1

2

3

4

5

**4+5**

**Picture S2** Fresh-frozen left hemiface after steps 4 and 5 of dissection. 1 = zygomatic branch of facial nerve; 2 + 3 = buccal branches of facial nerve; 4 = marginal branch of facial nerve; 5 = parotid duct


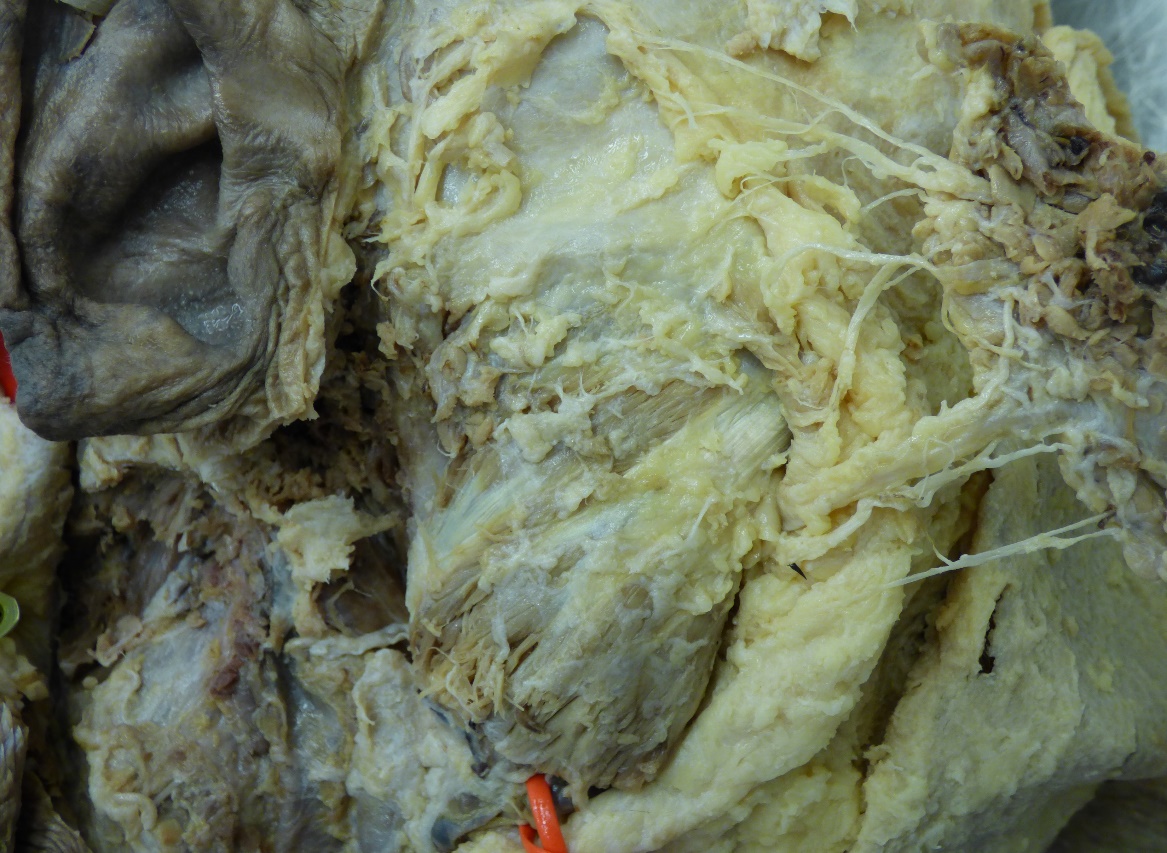


**7 + 8 + 9**

**5**

**1**

**2**

**3**

**6**

trunk (cut)

trunk (cut)

**anterior**

**Picture S3** Formalin-fixed right hemiface after steps 7, 8, and 9 of dissection. 1 = zygomatic branch of facial nerve; 2 + 3 = buccal branches of facial nerve; 5 = parotid duct; 6 = facial artery


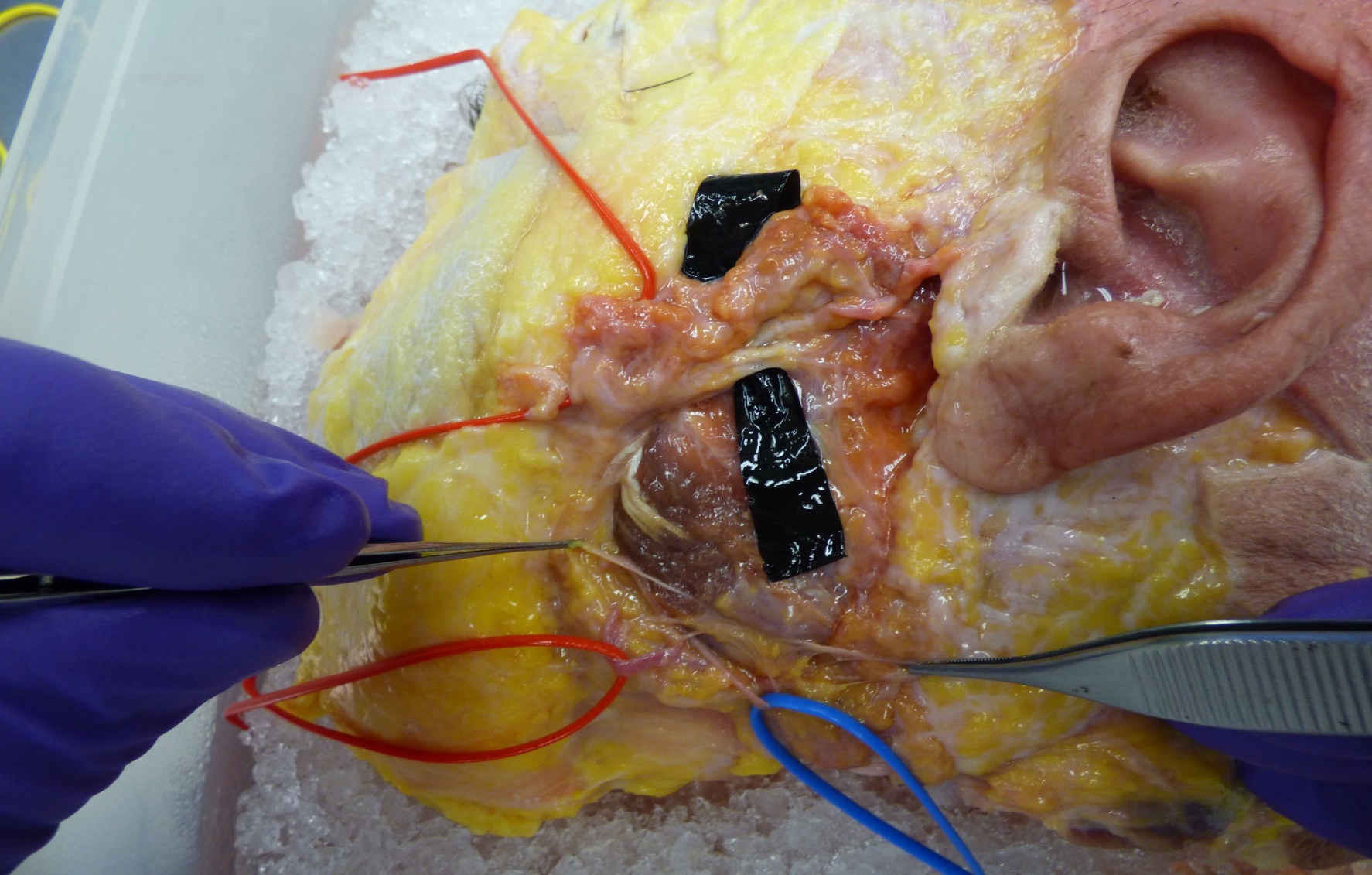


5

4

6

7

**8**

**Picture S4** Fresh-frozen left hemiface after step 8 of dissection. Note: cutting of main trunk of facial nerve not performed here. 4 = marginal branch of facial nerve; 5 = parotid duct; 6 = facial artery; 7 = facial vein


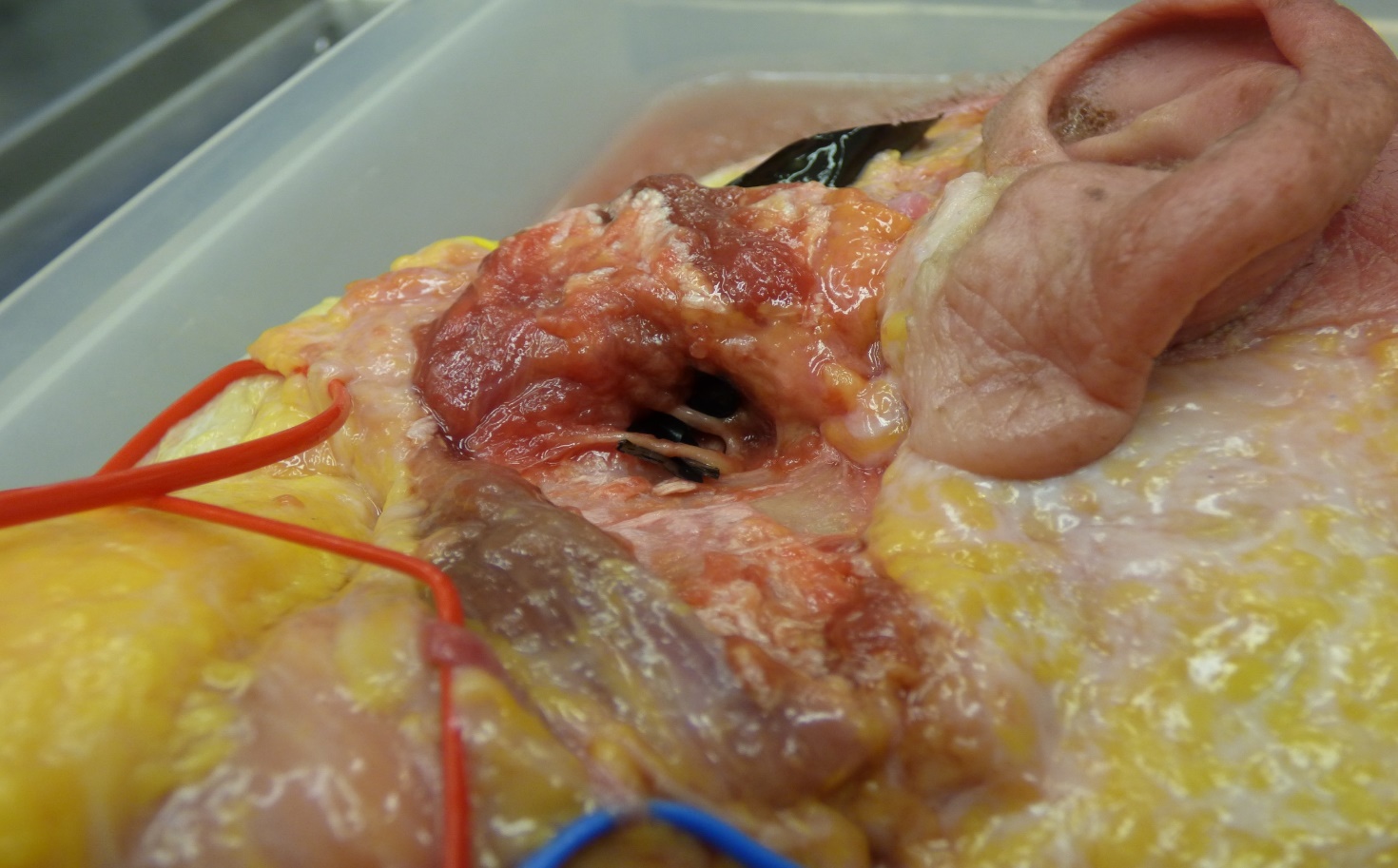


8a

8b

5

**9**

**Picture S5** Fresh-frozen left hemiface after step 9 of dissection. Note: masseter muscle is cut, dissected from the mandibula and superior part of the muscle, parotid gland, and facial nerve are reflected superiorly here. 5 = parotid duct; 8a = descending branch of masseteric nerve coming from the mandibular branch (CN V_3_) of the trigeminal nerve; 8b = transverse branch of the masseteric nerve coming from the mandibular branch of the trigeminal nerve


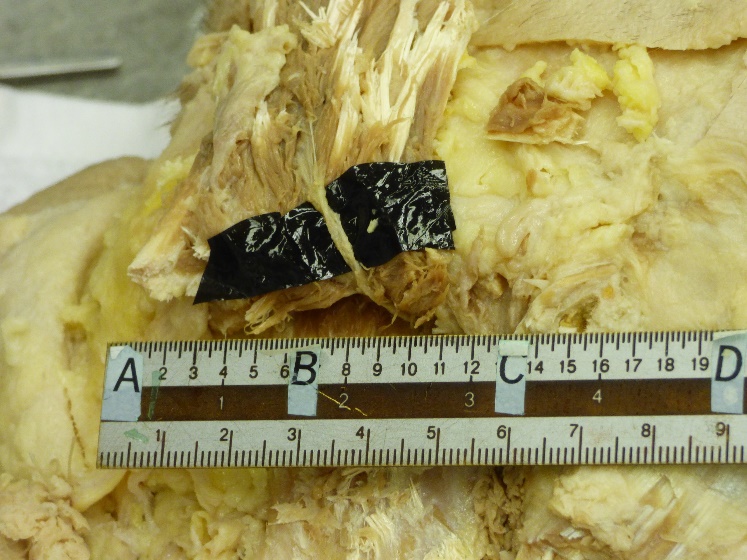

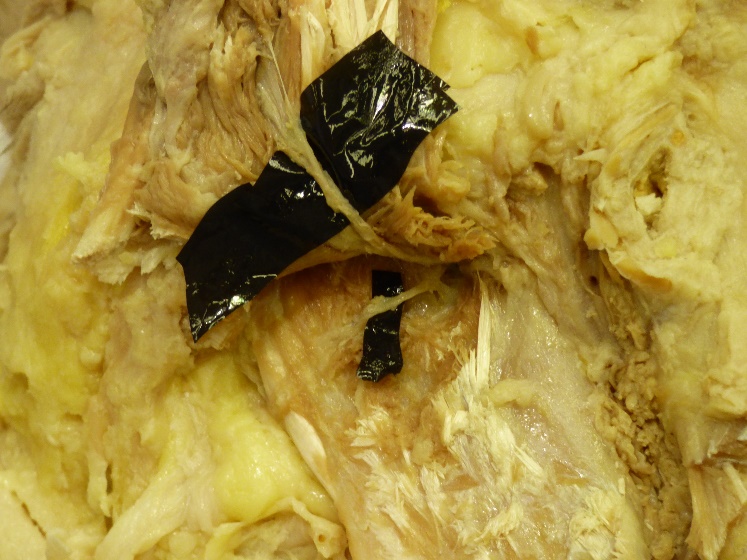


TMJ

meatus ext.

8a

8b

**9 + 10**

**9 + 10**

**anterior**

**anterior**

**Picture S6** Formalin-fixed left hemiface after step 9 and 10 of dissection. Note: both branches of masseteric nerve identified, master muscle detached and reflected cranially to zygomatic arch. 8a = descending branch of masseteric nerve coming from the mandibular branch (CN V_3_) of the trigeminal nerve; 8b = transverse branch of the masseteric nerve coming from the mandibular branch of the trigeminal nerve; TMJ = temporomandibular joint; meatus ext. = external acoustic meatus


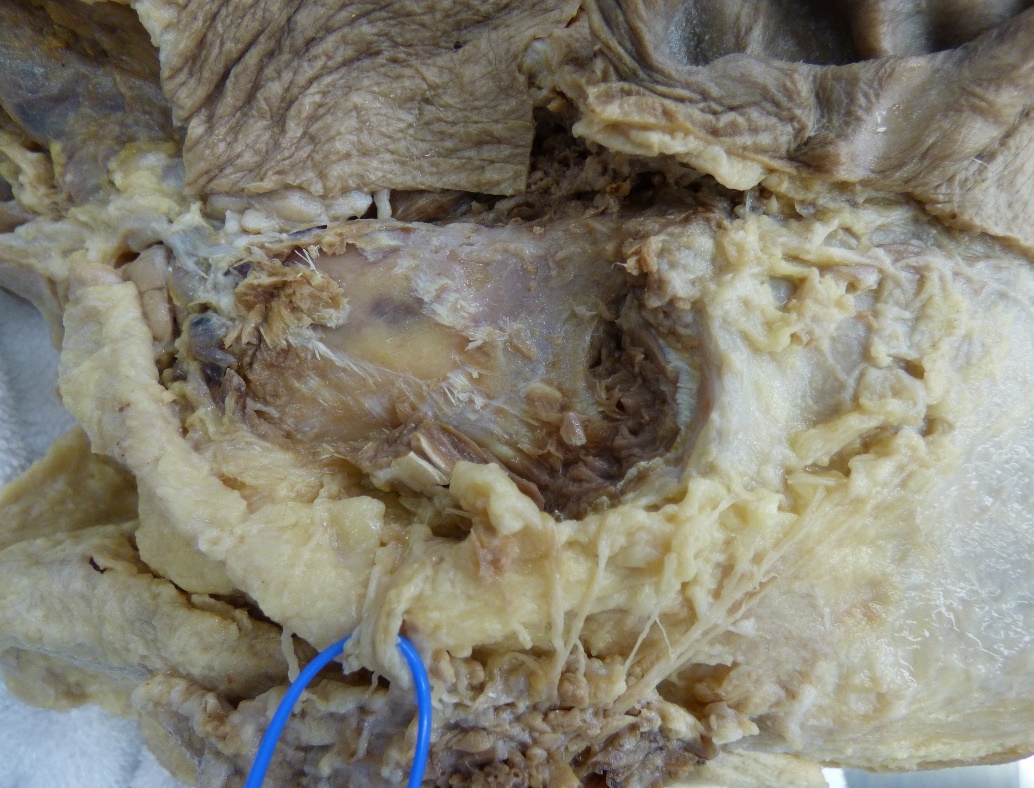


**1**

**2**

**5**

notch

masseter

strip

**10 + 11**

**anterior**

**Picture S7** Formalin-fixed right hemiface after steps 10 and 11 of dissection. Note: masseteric nerve not visible here and masseter muscle largely removed. 1 = zygomatic branch of facial nerve; 2 = buccal branches of facial nerve; 5 = parotid duct


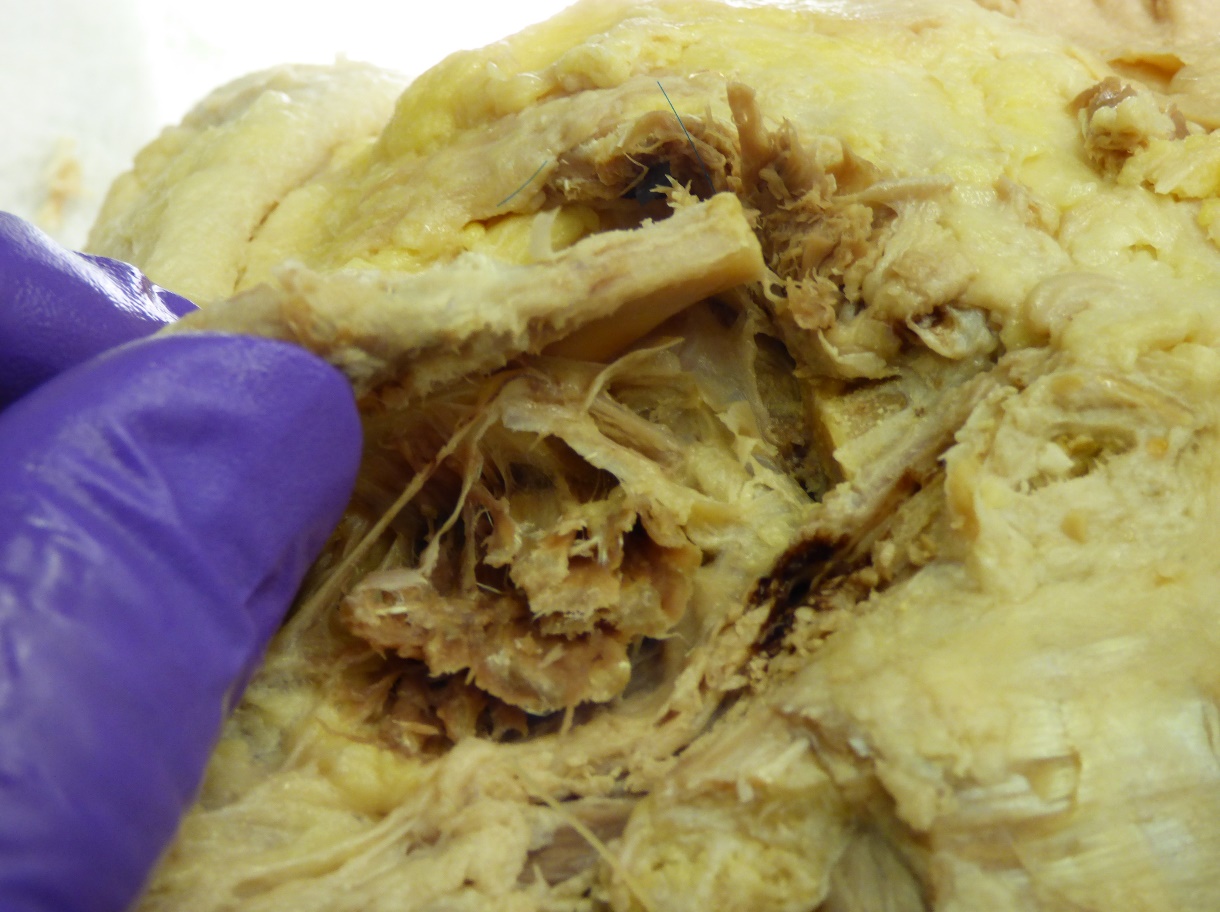


TMJ

meatus ext.

lat. pterygoid (cut)

med. pterygoid

(detached from mandible)

**14 + 15 + 16**

**anterior**

**Picture S8** Formalin-fixed left hemiface after steps 14, 15, and 16 of dissection. TMJ = temporomandibular joint; meatus ext. = external acoustic meatus


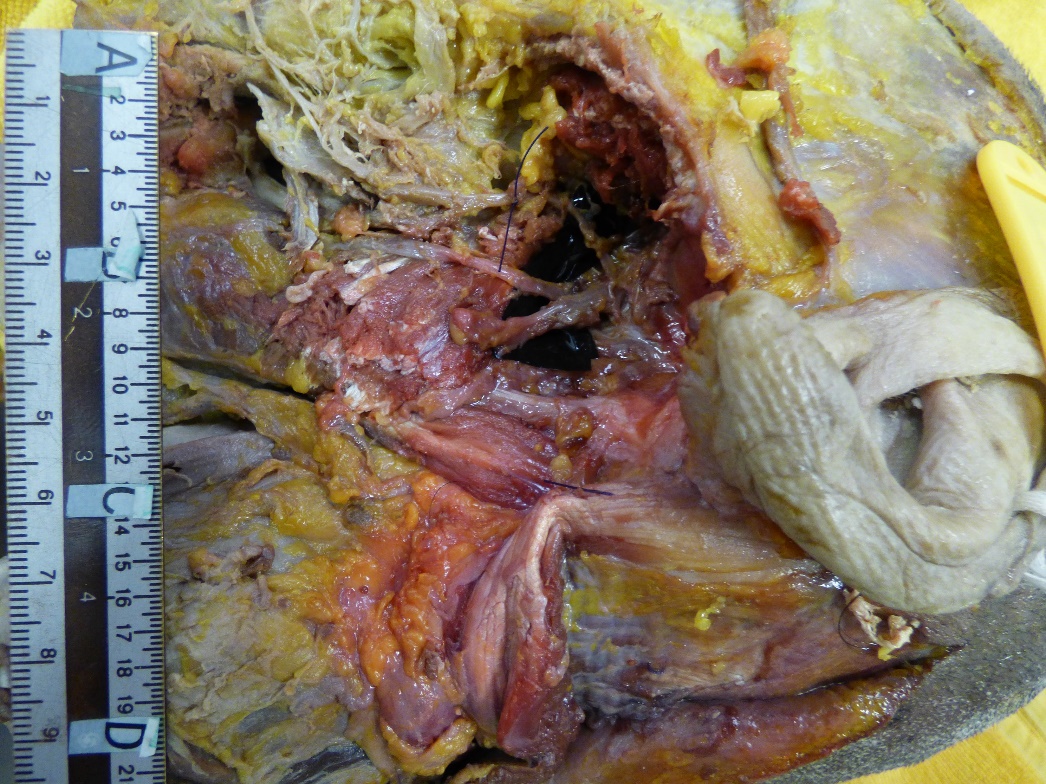


**anterior**

**17 + 18 + 19 + 20**

**Picture S9** Formalin-fixed left hemiface after steps 17, 18, 19, and 20 of dissection. Red circle = exit of the mandibular branch of the trigeminal nerve (CN V_3_) at the foramen ovale


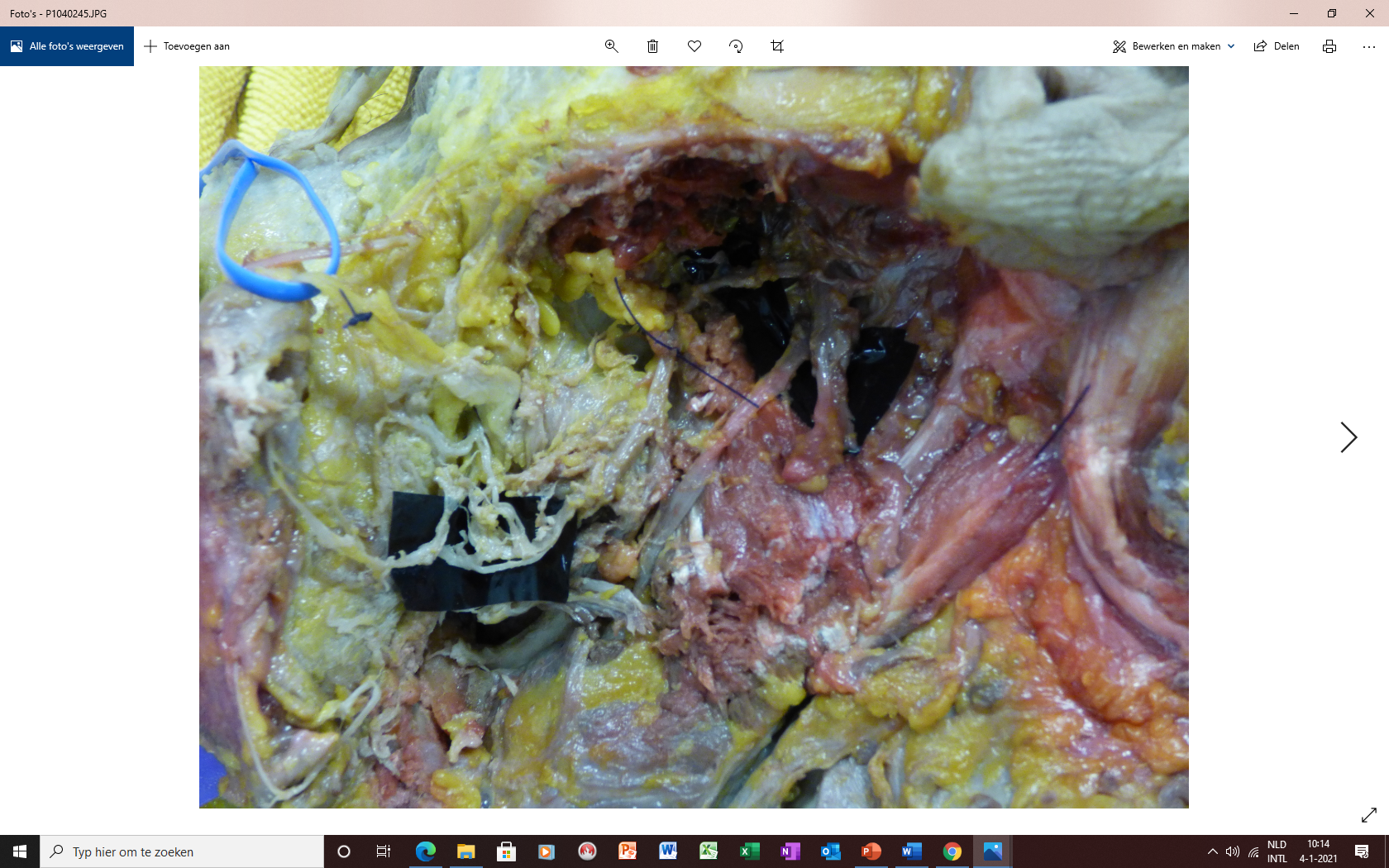


9

10

11

9

**anterior**

**21 + 22**

**Picture S10** Formalin-fixed left hemiface after steps 21 and 22 of dissection. 9 = buccal nerve originating from mandibular branch of trigeminal nerve (CN V_3_); 10 = lingual nerve; 11 = inferior alveolar nerve (cut); red circle = exit of mandibular branch of the trigeminal nerve at foramen ovale; red dashed line box = part of specimen enlarged in Picture S11

4

4

4

5

9

10

9


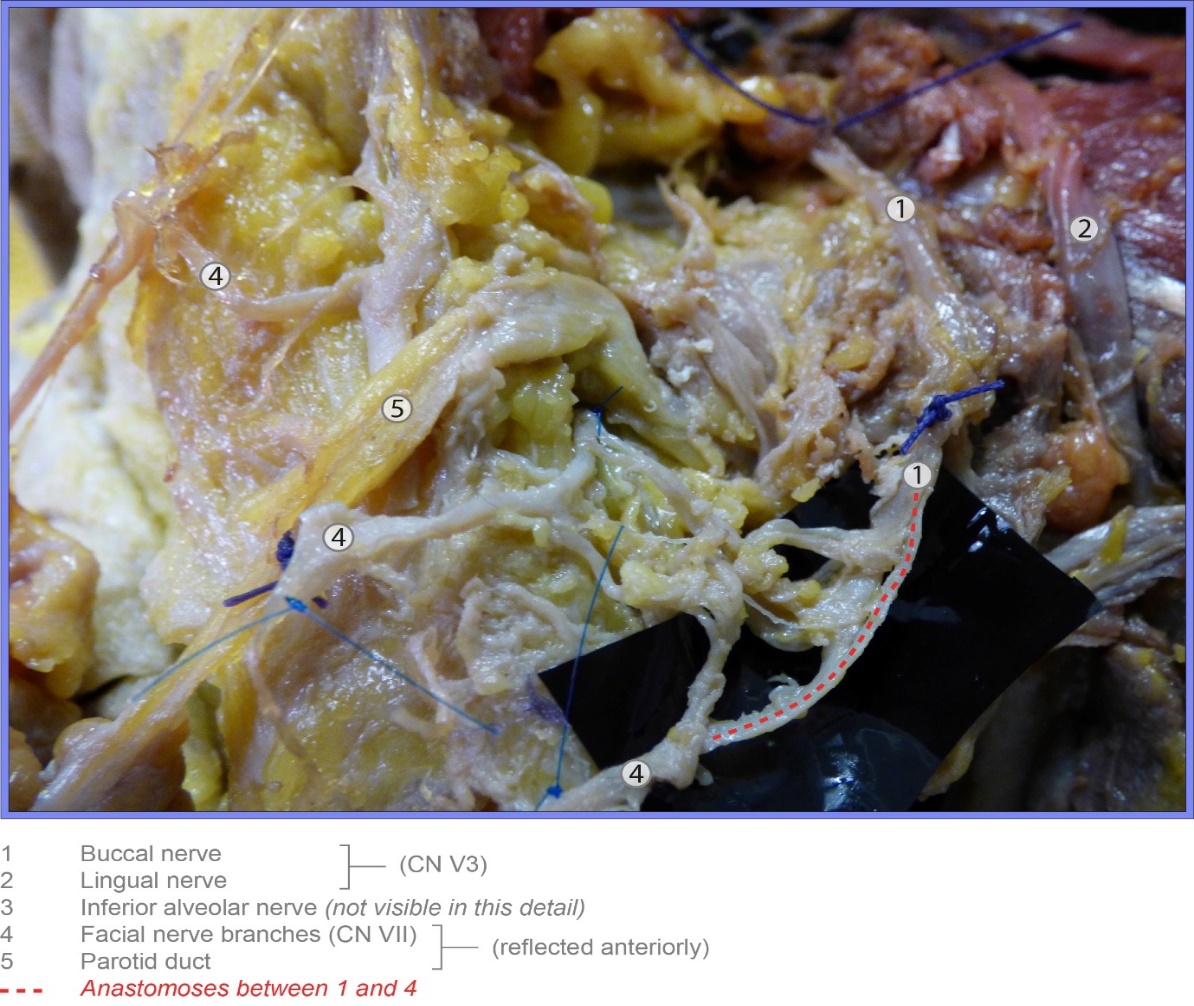


**anterior**

**21 + 22**

**Picture S11** Formalin-fixed left hemiface after steps 21 and 22 of dissection. Enlargement of red dashed line box in Picture S10. 4 = facial nerve branches (indicated with thin blue wire/knot); 5 = parotid duct; 9 = buccal nerve (indicated with thick blue wire/knot); 10 = lingual nerve; red dashed line is anastomosis between buccal nerve and facial nerve branch


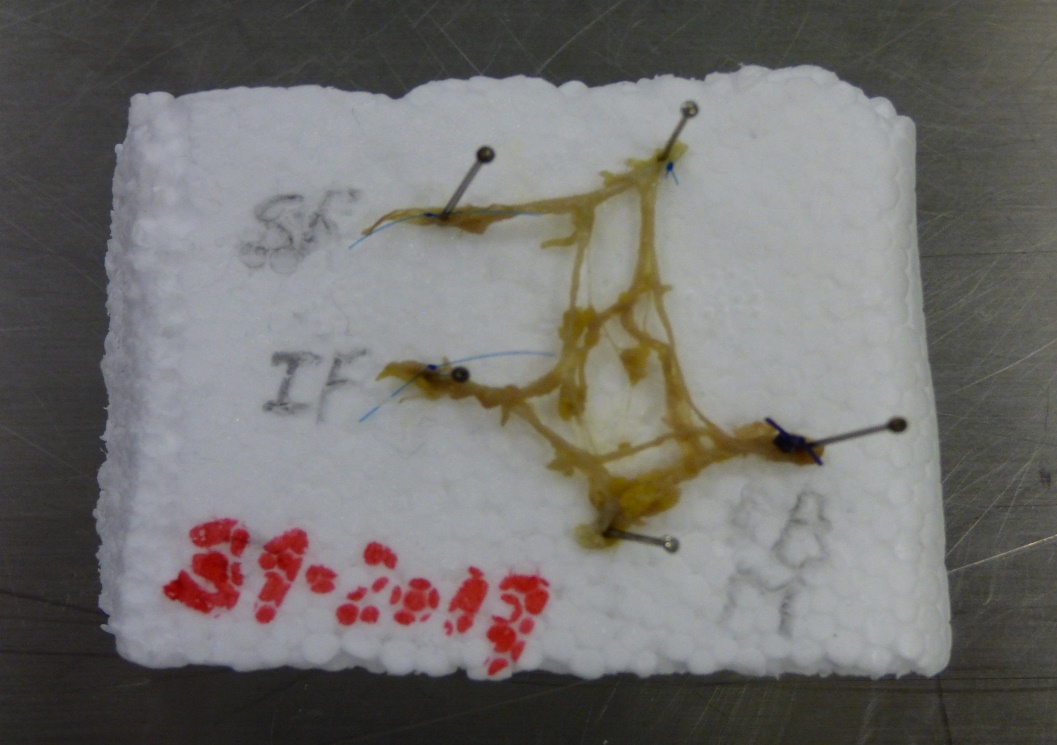


Superior facial VII

Inferior facial VII

Buccal V3

**Picture S12** Preparation of en bloc resection of facial and trigeminal nerve anastomoses on tempex for fixation.
